# Diverse presentations of cutaneous mosaicism occur in CYLD cutaneous syndrome and may result in parent-to-child transmission

**DOI:** 10.1016/j.jaad.2019.05.021

**Published:** 2019-12

**Authors:** Majid Arefi, Valerie Wilson, Siobhan Muthiah, Simon Zwolinski, Dalvir Bajwa, Paul Brennan, Katie Blasdale, David Bourn, John Burn, Mauro Santibanez-Koref, Neil Rajan

**Affiliations:** a Institute of Genetic Medicine, Newcastle University, Newcastle upon Tyne, United Kingdom; b Clinical Genetics, Centre for Life, Newcastle upon Tyne, United Kingdom; c Department of Dermatology, Royal Victoria Infirmary, Newcastle upon Tyne, United Kingdom

**Keywords:** Brooke-Spiegler syndrome, *CYLD*, CYLD cutaneous syndrome, genetic counseling, genetic testing of the skin, mosaicism, parent-to-child transmission, CCS, CYLD cutaneous syndrome, LOH, loss of heterozygosity, Mb, megabase, NGS, next-generation sequencing, PCR, polymerase chain reaction, PGD, preimplantation genetic diagnosis, SNP, single-nucleotide polymorphism

## Abstract

**Background:**

Clusters of rare cylindroma or spiradenoma tumors are a recurrent clinical presentation, yet conventional genetic testing results in individuals with these tumors are frequently normal.

**Objective:**

To determine if genetic mosaicism accounts for such cases.

**Methods:**

A study of 6 cases from a series of 55 patients who met criteria for diagnostic gene testing for pathogenic *CYLD* variants over a 5-year period (2012-2017) was performed. A novel genetic assay was used to study DNA from peripheral blood leukocytes and, where possible, matched skin and tumor tissue.

**Results:**

Two patients had mosaic pathogenic *CYLD* variants in both the blood and skin. One of these patients transmitted a pathogenic variant to her daughter, and we report the novel phenotype of a contiguous gene deletion syndrome involving *CYLD*. Two patients had recurrent pathogenic variants in skin tumors from a single cluster but none detectable in the blood.

**Limitations:**

The remaining 2 patients had clinical features of mosaicism, but these cases were not solved with the assays used because of a lack of access of fresh tumor tissue.

**Conclusion:**

Genetic mosaicism should be considered in patients presenting with clustered cylindromas, because this may inform genetic testing and counseling of these patients.

## Graphical abstract

**Figure:**
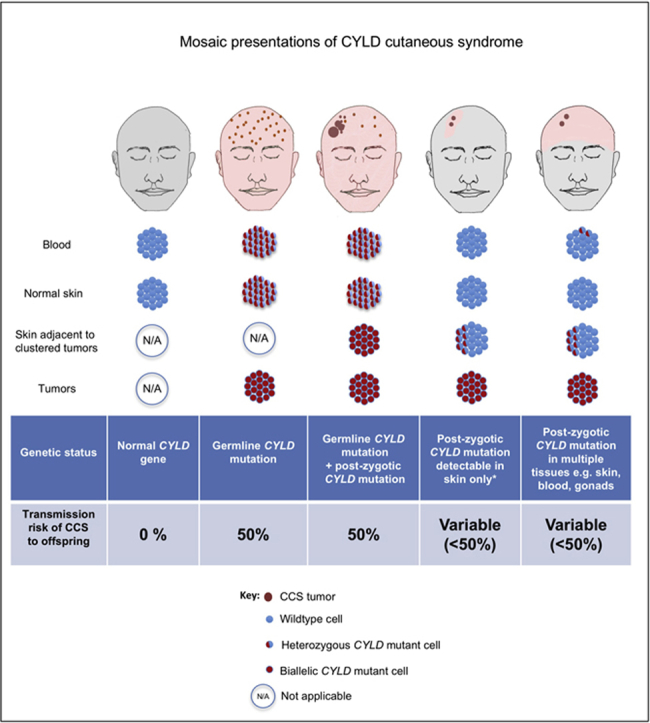

**Capsule Summary**

• Clusters of asymmetric cylindromas are a clinical indicator of underlying genetic mosaicism.
• In such patients who lack a family history, genetic testing of skin tumors is necessary. This is relevant for genetic counseling because these patients can transmit a more severe form of the disease to their children.

CYLD cutaneous syndrome (CCS), also called Brooke-Spiegler syndrome, is characterized by the development of multiple adnexal skin tumors that can include cylindromas, spiradenomas, and trichoepitheliomas.^1^, ^2^, ^3^ Genetic linkage studies suggest that CCS is a single-locus disease,^4^ with exonic germline pathogenic *CYLD* variants shown in up to 85% of cases.^5^ Some cases deemed mutation-negative by Sanger sequencing are associated with large rearrangements,^6^ intronic variants affecting splicing,^7^ and large deletions^8^ in isolated case reports. However, the remaining cases are unexplained, presenting a challenge for genetic counseling of affected individuals.

Postzygotic mutation resulting in mosaicism may account for some of these unexplained cases. It is suggested clinically by affected individuals who develop CCS tumors in an asymmetric distribution, typically clustered or in a linear array on the scalp or torso.^9^, ^10^, ^11^, ^12^, ^13^, ^14^ Postzygotic mutation of genes affecting keratinocytes during embryonic development can result in skin conditions that follow Blaschko's lines.^15^ CCS tumors have been reported to manifest in a Blaschko-linear pattern^14^; however, mosaic pathogenic *CYLD* variants have not been previously shown in such cases.^9^

CCS is a dominantly inherited skin tumor syndrome.^2^ *CYLD* encodes a ubiquitin hydrolase enzyme involved in the posttranslational modification deubiquitination.^16^, ^17^, ^18^, ^19^ By removing ubiquitin chains from relevant protein substrates, CYLD normally negatively regulates cell signaling pathways that are implicated in tumor formation, such as nuclear factor κB, Wnt, and transforming growth factor–β.^2^ In patients with familial CCS, germline heterozygous pathogenic *CYLD* variants reported within the catalytic residues of *CYLD* are frequently truncating,^20^ predicting loss of function. CCS tumors typically have a “second hit” that affects the remaining normal copy of *CYLD*, resulting in loss of heterozygosity (LOH).^21^ We posited that postzygotic *CYLD* mutations could occur during embryonic development, with subsequent LOH in affected cells leading to mosaic presentations of CCS tumors.

In this study, we investigated this hypothesis in patients with clustered tumors who fit clinical criteria for diagnostic testing of *CYLD*^22^ but lacked a pathogenic *CYLD* variant detectable via Sanger sequencing of coding exons in a peripheral blood sample. By using novel genetic assays to study blood and tumor tissue in these patients, we show the existence of postzygotic mutations resulting in mosaicism in patients with clusters of CCS tumors. We also document transmission from a parent with mosaic CCS to a child, underscoring the clinical relevance of obtaining a genetic diagnosis in these cases.

## Methods

### Ethics

Regulatory approvals were sought and obtained from a human subjects ethics review board (National Research Ethics Service Committee North East-Tyne and Wear, reference no. 14/NE/1080;06/1059).

### Sample collection

Consenting patients supplied blood samples for DNA analysis. Fresh-frozen tissue or RNAlater-stored material (RNAlater solution, Life Technologies, Grand Island, NY) was also available in some cases.

### DNA and RNA extraction

Genomic DNA was extracted from fresh-frozen tissue and peripheral blood with the DNeasy Tissue kit (Qiagen, Hilden, Germany) according to the manufacturer's protocol. RNA was extracted from trial samples and control samples as previously described.^23^

### Genetic assays

We used a long-range polymerase chain reaction (PCR) assay coupled with next-generation sequencing (NGS) to investigate the entire 56-kilobase genomic locus of *CYLD* with an average on-target sequencing depth of greater than 2000× (here after referred to as long-range PCR [*LR-PCR*]). Briefly, 8 amplicons ranging from 6 to 8 kilobase in size were designed to cover the promoter, coding exons, and introns of *CYLD*. Primer sequences and PCR conditions are available on request. Findings were validated by using NGS of affected amplicons or, in selected cases in which the minor allele frequency was greater than 0.2, Sanger sequencing of amplicons. In 2 tumors for case 4, RNA sequencing data were used for pathogenic *CYLD* variant detection. In 3 tumors for case 4, single-nucleotide polymorphism (SNP) arrays were used to detect large deletions.

## Results

**Fig 1:**
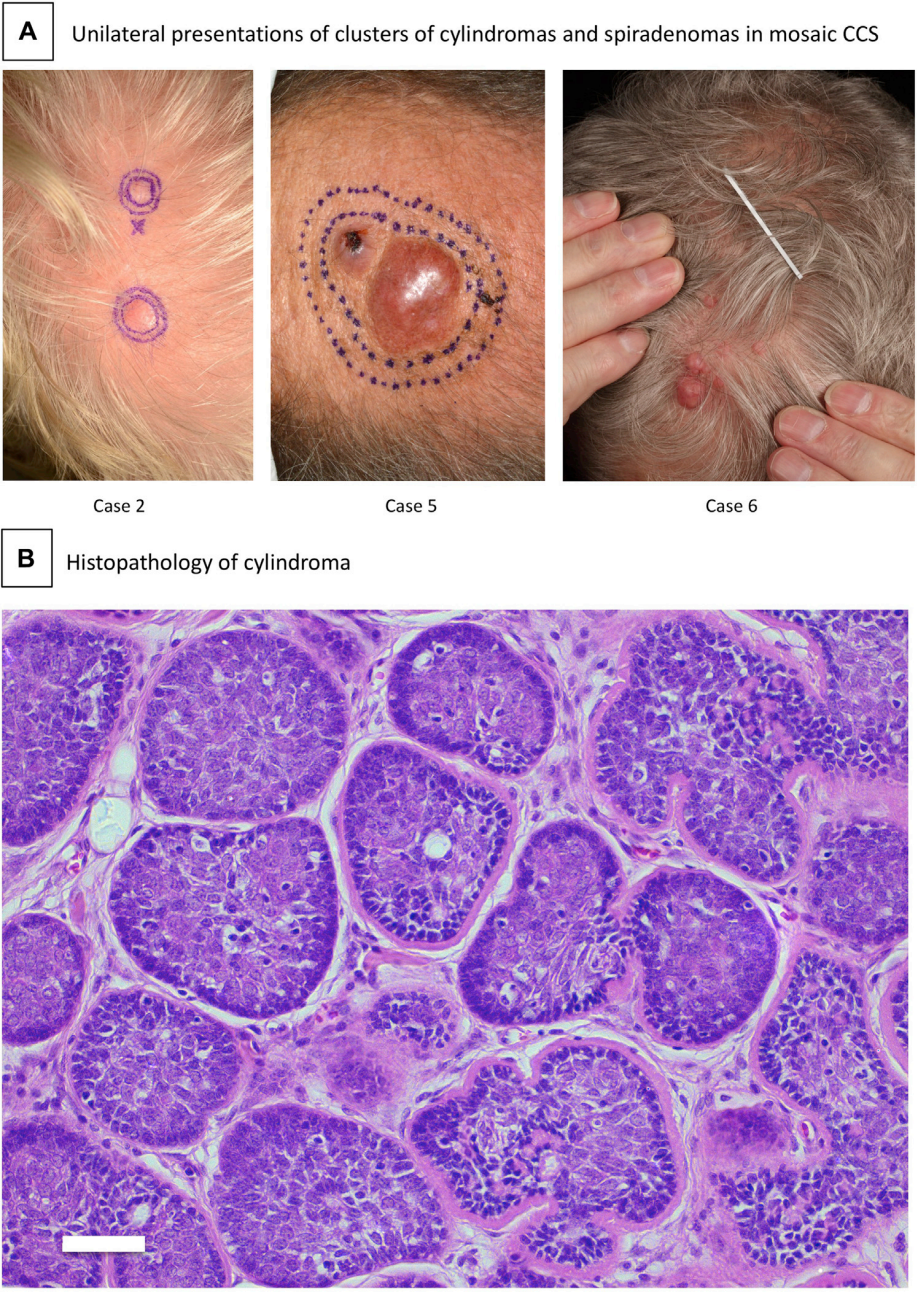
Mosaic CYLD cutaneous syndrome. **A**, Localized clusters of cylindroma or cylindrospiradenoma shown, from left to right, in cases 2, 5, and 6. **B**, Histopathology of cylindroma (hematoxylin-eosin stain; original magnification: 20). White scale bar indicates 50 μm.

We reviewed 55 patients who met criteria^22^ for diagnostic gene testing for pathogenic *CYLD* variants. After Sanger sequencing of coding exons of *CYLD* from peripheral leukocyte DNA, 39 cases were deemed to carry pathogenic *CYLD* variants. From the 16 mutation-negative cases, we included 5 patients with a single localized cluster of CCS tumors and 1 patient with bilateral clusters, because the latter presentation is also compatible with mosaicism (Table I and Fig 1, *A* and *B*). The patients with localized clusters had 2 to 8 tumors, which ranged from 3 mm to 3 cm in diameter. The clusters were 3 to 8 cm in size, with constituent lesions in direct apposition or up to 4 cm apart. Five of the 6 patients with clustered tumors had no family history of CCS. In the 10 remaining patients without clustered tumors, 3 had a positive family history result.

**Table I:** Clinical cases of mosaic CCS deemed mutation-negative after Sanger sequencing of coding exons

Case	Age at onset of skin tumors, years	Family history	Phenotype	Blood (NGS or array)	Skin tumor	*CYLD* tumor variant and ACGS classification (LOH)∗
1	30s	NEG	Single localized cluster of cylindromas (n > 5)	8% mutant reads	2 tumors, truncating variant exon 19	c.2806C>T p.(Arg936*) Pathogenic
2	70s	NEG	Single localized cluster of cylindromas (n = 2)	Normal NGS results	2 tumors, 25-bp deletion exon 19	(c.2499_2524del p.(His833Glnfs*48) Likely pathogenic
3	30s	NEG	Single localized cluster of cylindromas (n = 4)	Normal NGS results	2 tumors, frameshift variant exon 11	(c.1520_1527delinsCTGTACAGAA; p.(Glu507fs) Pathogenic
4	30s	NEG	Bilateral clusters of cylindromas; 3 tumors had features of cylindrospiradenoma (n > 20)	Normal NGS and array results	5 tumors, diverse single-nucleotide variants; recurrent 5.5-Mb deletion in tumors 1-3.	Tumor 1: c.1912G>T p.(Glu638*) Pathogenic Tumor 2: c.1808T>G p.(Leu603*) Pathogenic Tumor 3: c.1821dupA p.(Phe608Ilefs*7) Pathogenic Tumor 4: c.1112C>A p.(Ser371*) Pathogenic Tumor 5: c.2158G>A p.(Glu720Lys) Likely pathogenic
5	50s	NEG	Single localized cluster of cylindromas; biopsy results showed 1 tumor had features of cylindrospiradenoma (n = 3)	Normal NGS results	ND†	ND†
6	50s	POS	Single localized cluster of cylindromas (n = 8)	Normal NGS and array results	Not examined	Not examined

*ACGS*, Association for Clinical Genomic Science; *LOH*, loss of heterozygosity in all cases with reported variants; *ND*, no data; *NEG*, negative family history of cylindromas; *NGS*, long-range PCR targeting the *CYLD* locus coupled with next-generation sequencing; *POS*, positive family history of cylindromas.

∗ *CYLD* mutations are annotated according to RefSeq: NM_015247.

† Comprehensive analysis of all coding exons was not feasible because of the technical limitations of studying DNA derived from paraffin-embedded tissue.

### Mosaic *CYLD* point mutation in the blood and skin as a cause of asymmetric CCS

On LR-PCR, case 1 showed 8% mutant reads of a previously reported^24^ pathogenic variant in *CYLD* present in the blood. Analysis of tumor tissue from 2 samples from this patient by Sanger sequencing clearly showed this variant, present at a minor allele frequency of more than 80%, indicative of LOH. LR-PCR analysis did not show a mosaic pathogenic *CYLD* variant in the blood in the other 5 cases.

### Mosaic *CYLD* mutation in the skin alone as a cause of asymmetric CCS

In case 2 (Fig 1, *A*), 2 tumors developed at the age of 70 years and grew progressively on the left side of the vertex of the scalp. Both tumor samples showed an identical 25–base pair deletion in exon 19 of *CYLD*, with LOH. We also analyzed interlesional normal-appearing skin using the same approach and detected the same pathogenic *CYLD* variant in a very low percentage of sequence reads in normal-appearing skin (18/5886 reads; 0.31%), and, notably, this variant was not evident in a peripheral leukocyte and a buccal DNA sample. Case 3 reported multiple localized tumors presenting from the 3rd decade. Two separate tumors were analyzed, and an identical frameshift pathogenic *CYLD* variant in exon 11 with LOH of *CYLD* was detected in both tumors.

### Mosaic large deletions in the blood and skin as a cause of CCS

In case 4, 20 cylindromas had developed since the age of 30 years, with bilateral clusters seen on the forehead. Five distinct pathogenic *CYLD* variants were found in 5 tumors, with 1 in each tumor (Table I). Results of SNP array analysis of the blood were normal, ruling out a heterozygous germline deletion. However, because SNP arrays are unable to detect low-level mosaicism in blood, we performed SNP arrays on DNA from 3 of the 5 tumors, in which such mosaic deletions would be clonally amplified. This showed an identical 5.5-megabase (Mb) deletion encompassing *CYLD* and 23 other genes in all 3 tumors at a minor allele frequency of approximately 80%.

### Transmission of mosaic CCS from parent to child and the phenotype of a contiguous deletion syndrome involving *CYLD*

**Fig 2:**
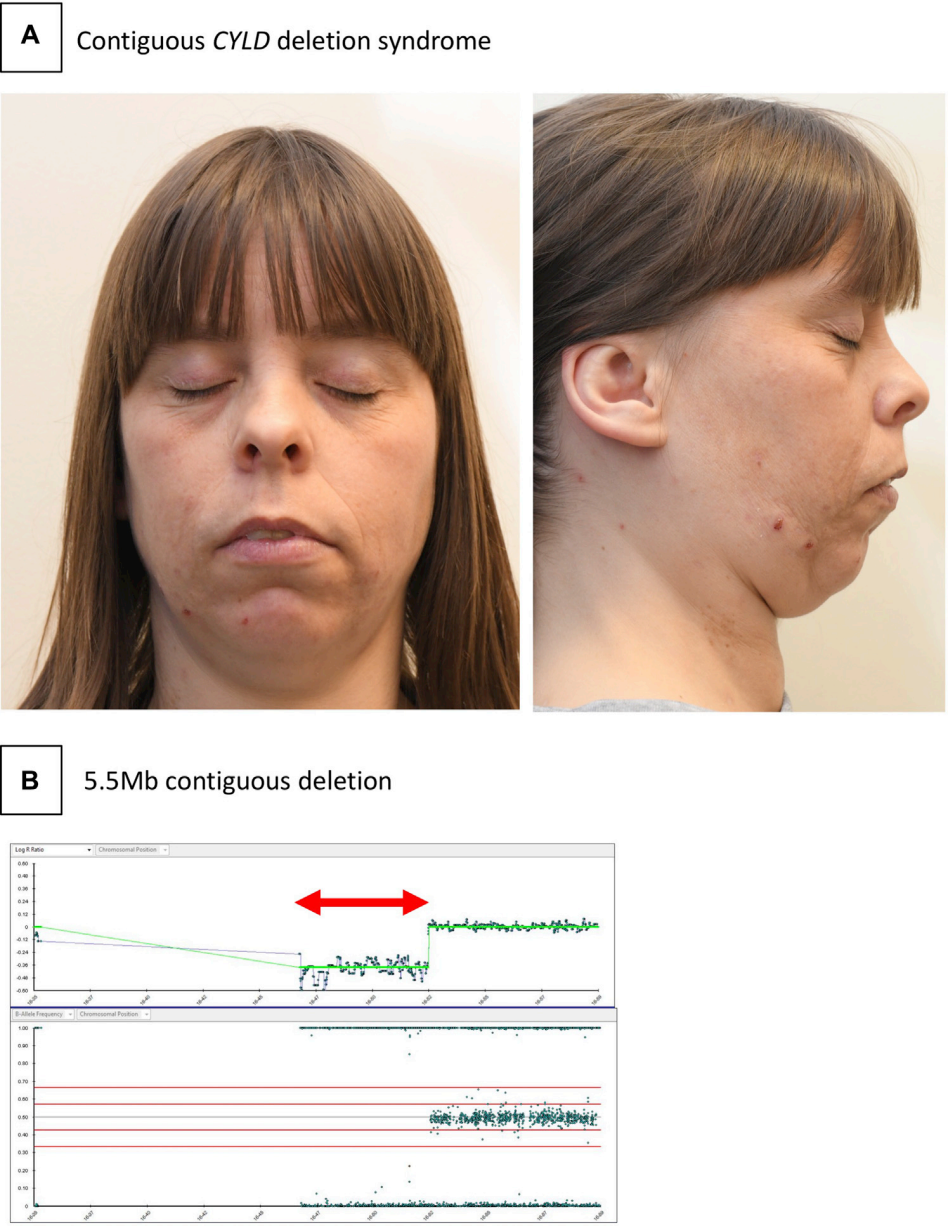
CYLD contiguous deletion syndrome. **A**, The facial gestalt of the daughter with a germline contiguous gene deletion involving *CYLD*. **B**, The 5.5-Mb deletion, indicated within the double-ended red arrows, was validated in the child as present in a heterozygous state and involved *CYLD* and 23 additional genes.

We proceeded to investigate the daughter of case 4 (Fig 2, *A*), who had mild intellectual disability and a single kidney from birth but did not have cylindromas when examined in her 30s. She had mild dysmorphic facial features, including anteverted nares, a long philtrum, retroverted small ears, and mild retrognathia. SNP array analysis of peripheral lymphocyte DNA showed the presence of the heterozygous germline 5.5-Mb deletion seen in her mother's tumors (Fig 2, *B*). Interrogation of the DECIPHER database (a public database of DNA microarray and sequencing data of children with developmental disorders) showed an additional 7 cases who carried a deletion including *CYLD* in the germline.^25^ Three of these patients had renal hypoplasia, 5 had intellectual disability, and 1 had a skin tumor. Additional recurrent features reported include abnormality of the pinnae, anal atresia, and hypospadias in males.

### Clinical features of mosaicism in unsolved cases

Case 5, who did not have a family history of CCS, reported a single tumor appearing on the scalp in his 50s and 2 more tumors appearing on the same side of the scalp when he was in his 60s. The clinical presentation was consistent with a postzygotic mutation affecting *CYLD*. Only paraffin-embedded tumor tissue was available, precluding the use of LR-PCR. Sanger sequencing of coding exons was successful in 40% of coding exons but inconclusive in the remainder. Case 6 had a single localized cluster of tumors (Fig 1, *A*) and a family history of CCS affecting 3 generations. The clinical presentation was consistent with the combination of a germline and a postzygotic mutation affecting *CYLD*. A germline deletion was excluded by using an SNP array. Tumor tissue was not available for genetic analysis in this case.

## Discussion

In this study, we found clustered cylindromas or cylindrospiradenomas in 6 of 16 mutation-negative cases of CCS; in 4 of these, we found novel molecular evidence of genetic mosaicism. The rarity of CCS tumors in the general population makes the development of asymmetric clustered CCS tumors (including trichoepitheliomas) a flag that should lead the physician to consider a diagnosis of mosaic CCS. In CCS, in which tumors predominantly manifest on the scalp, asymmetric clustered presentations may be masked by hair, and careful clinical examination is warranted.

Patients with mosaic CCS should have their genetic testing tailored depending on their family history. Patients with a postzygotic mutation alone lack a family history and are likely to have a negative result from Sanger sequencing of *CYLD* in a blood sample (unless the level of leukocyte mosaicism is >40%). A negative result should prompt analysis of DNA from at least 2 skin tumor samples, optimally from non–paraffin-embedded tissue. The presence of a recurring pathogenic *CYLD* variant detectable in multiple skin tumors within this cluster supports mosaicism, and this information can be used in genetic testing of children or in preimplantation genetic diagnosis (PGD). A caveat is that mosaic tumors may occur in more than 1 cluster and can have a bilateral distribution, as shown by case 4. Hence, cases deemed mutation-negative by Sanger sequencing with bilateral clustered tumors may also benefit from this approach, particularly if they lack a parental history of CCS. It should also be considered that some mosaic cases with high levels of mosaicism may mimic the phenotype of germline cases.

Patients with a parental history of CCS and an asymmetric cluster of tumors are expected to have a postzygotic second-hit *CYLD* mutation within these lesions in addition to the familial germline *CYLD* variant.^26^ Sanger sequencing of blood leukocyte DNA alone can yield an answer in these patients, unless they have a genetic mutation that prevents PCR amplification of the mutant allele, such as a large deletion, inversion, or rearrangement involving *CYLD*. As the cost of NGS decreases, whole-genome sequencing of blood is likely to be increasingly adopted in diagnostic settings. This has the advantage of overcoming the limitations of PCR, with the potential to detect mosaicism, providing there is sufficient coverage and sequencing depth of the *CYLD* locus.

A further informative point in case 4 is the finding of 5 distinct pathogenic *CYLD* variants in 5 tumors, with a large deletion involving the other allele. This finding suggests that the complete deletion of *CYLD* alone may be insufficient for the formation of cylindromas, which is reminiscent of phenotypic findings in *CYLD* mouse models, in which homozygous *CYLD* deletion results in a normal mouse at birth^27^; however, transgenic mice expressing homozygous truncating patient mutations die at birth.^28^ Future work to study the differential effects of deletions and truncating mutations in *CYLD* on tumorigenesis may help explain our research findings in case 4.

The importance of finding the pathogenic *CYLD* variant in mosaic cases is emphasized by our report of transmission of a pathogenic *CYLD* variant from a mosaic parent to the germline of a child. In this case, and in others recorded in DECIPHER, the clinical phenotype of the heterozygous contiguous gene deletion is more complex and severe than the mosaic form. Notably, skin tumors are not frequently reported in the DECIPHER series, which may reflect the early age at which these cases are studied, typically in the first years of life. We suggest that the presentation of cylindroma, or a family history of cylindromas, together with intellectual disability and/or renal abnormalities should alert the clinician to consider a contiguous gene-deletion syndrome. The risk to a mosaic CCS parent carrying a postzygotic mutation of conceiving a germline-affected child cannot, currently, be accurately estimated, although DNA analysis of sperm in males may help. Otherwise, PGD, or other prenatal diagnostic strategies, may be considered in mosaic individuals who plan to start a family, once the pathogenic *CYLD* variant is known. The selection of PGD, which carries attendant risks, may be influenced by the level of mosaicism in the affected parent.

Our method has limitations. The LR-PCR assay we report is able to detect mosaic exonic, intronic, and promoter changes within the *CYLD* locus, which is an advantage over Sanger sequencing of coding exons. However, it is unable to detect large deletions, gene fusions, and inversions, and these explanations may account for the lack of a molecular diagnosis in case 6. We determined 3 cases to have mosaicism in the skin alone using our assay by studying blood DNA; however, we cannot exclude the possibility of rare mutant cells that are below the threshold of detection of our assay. As such, there is still a theoretical risk of transmission, albeit small, that can be excluded only by PGD. We did not seek to determine the lower limit of detection of our assay in this study, and this limit would be required to implement this assay in a diagnostic setting.

## Conclusions

Our findings have pragmatic implications for genetic investigation of mutation-negative CCS cases. The selection of assays will vary from center to center and be influenced by resource and sequencing infrastructure but must be able to detect mosaicism in mutation-negative CCS cases. Our findings are relevant to other skin tumor syndromes in which linear or clustered tumors such as basal cell carcinoma may occur.^29^, ^30^ We emphasize the need to analyze affected tumor tissue^31^ from these patients if blood proves to be mutation-negative to identify genetic mosaicism, which confers a risk of transmission to offspring.
